# Supplementary figures and images for: Auditory chain reaction: Effects of sound pressure and particle motion on auditory structures in fishes
Source: PLoS One. 2020 Mar 27;15(3):e0230578. doi: 10.1371/journal.pone.0230578 (PMC7100961; doi:10.1371/journal.pone.0230578)

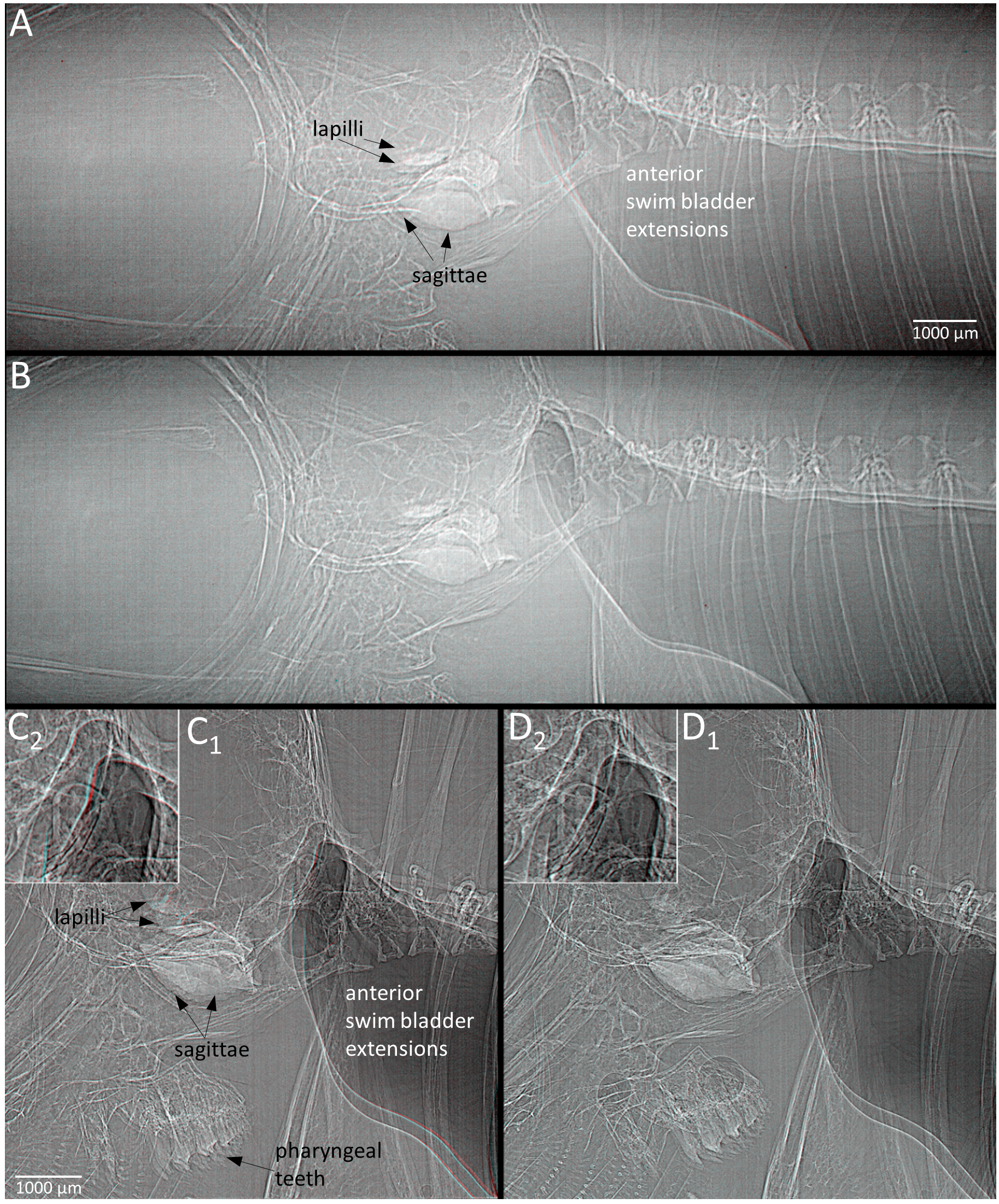

Supplement: S1 Fig — Overlays of averaged maximum (red) and minimum (green) positions indicate the motion of otoliths (lapilli, sagittae) and the walls of the anterior swim bladder extensions due to the stimulus presentation. (A) and (C) depict the structures during the in phase (0°) condition of the sound stimulus, whereas (B) and (D) represent the out of phase (180°) condition. (C2) vs. (D2) illustrates that the swim bladder walls show distinct motion (shakers driven in phase) as opposed to no oscillation (shakers driven out of phase). Structures outlined in red and green indicate motion, whereas uniformly gray, white or black structures point to no or weak movement during sound presentation. The overlays in (A-B) represent an individual (SL = 51 mm) studied at ID17 (pixel size 6.1 µm, frame rate 98.9954 fps) and those in (C-D) illustrate a specimen (SL = 48 mm) imaged at ID19 (pixel size 3.67 µm, frame rate 198.02 fps). (TIF) [file pone.0230578.s008.tif]

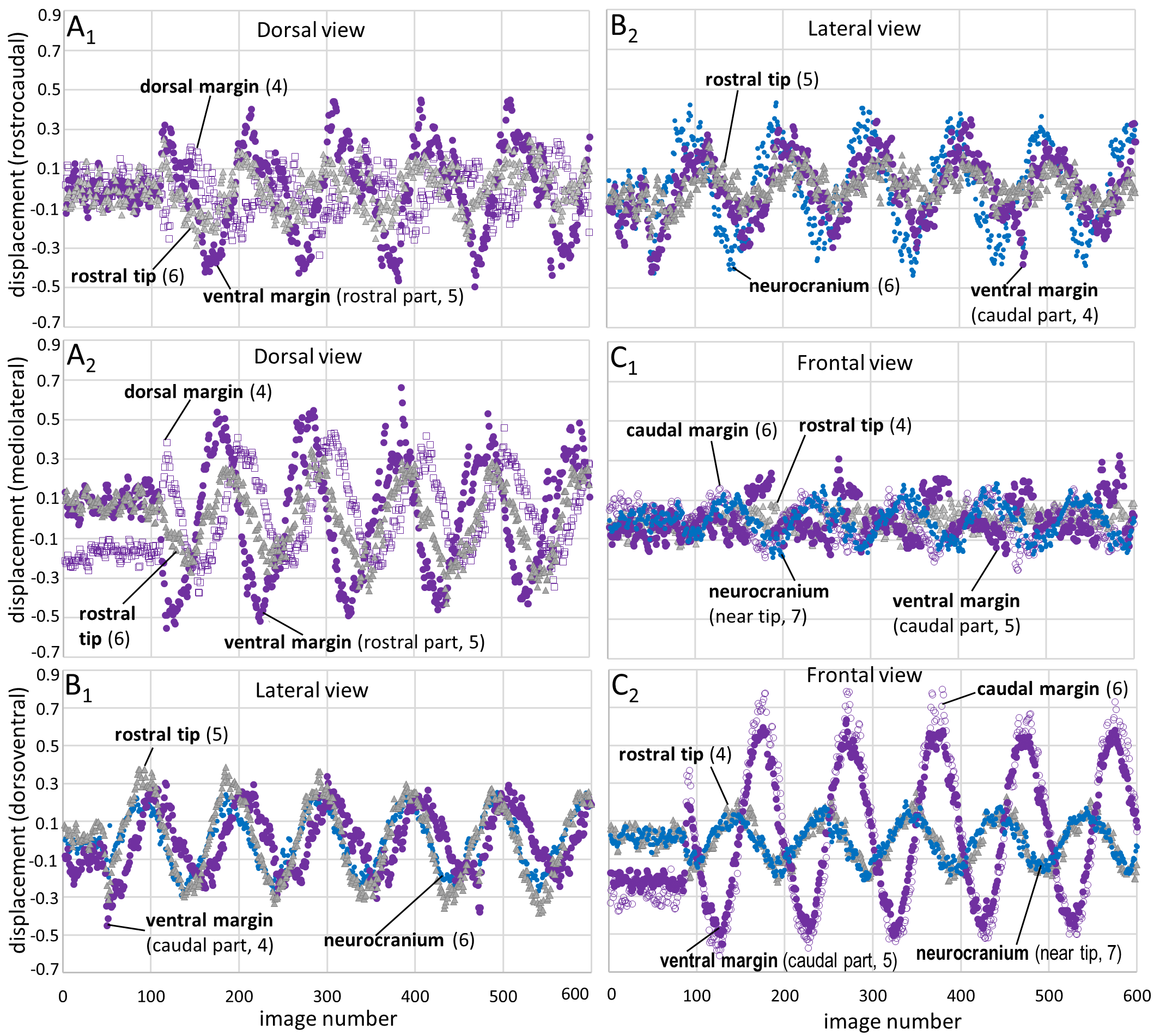

Supplement: S2 Fig — Motion patterns are shown in (A1-2) dorsal, (B1-2) lateral, and (C1-2) frontal views (pixel size 3.67 µm, ID19) when the fish was subjected to the in phase (0°) condition. The clear phase shift between different parts of the sagitta in all plots except in (B2) indicates the tilting movement of this otolith during sound presentation. In (C1), the rostral tip of the sagitta shows less displacement than the ventral and posterior margins and moves in phase with the adjacent part of the neurocranium, which is also seen in (B1). The respective landmark number is given in parenthesis (see also Figs 5B1, 6B1 and 8B1). (TIF) [file pone.0230578.s009.tif]

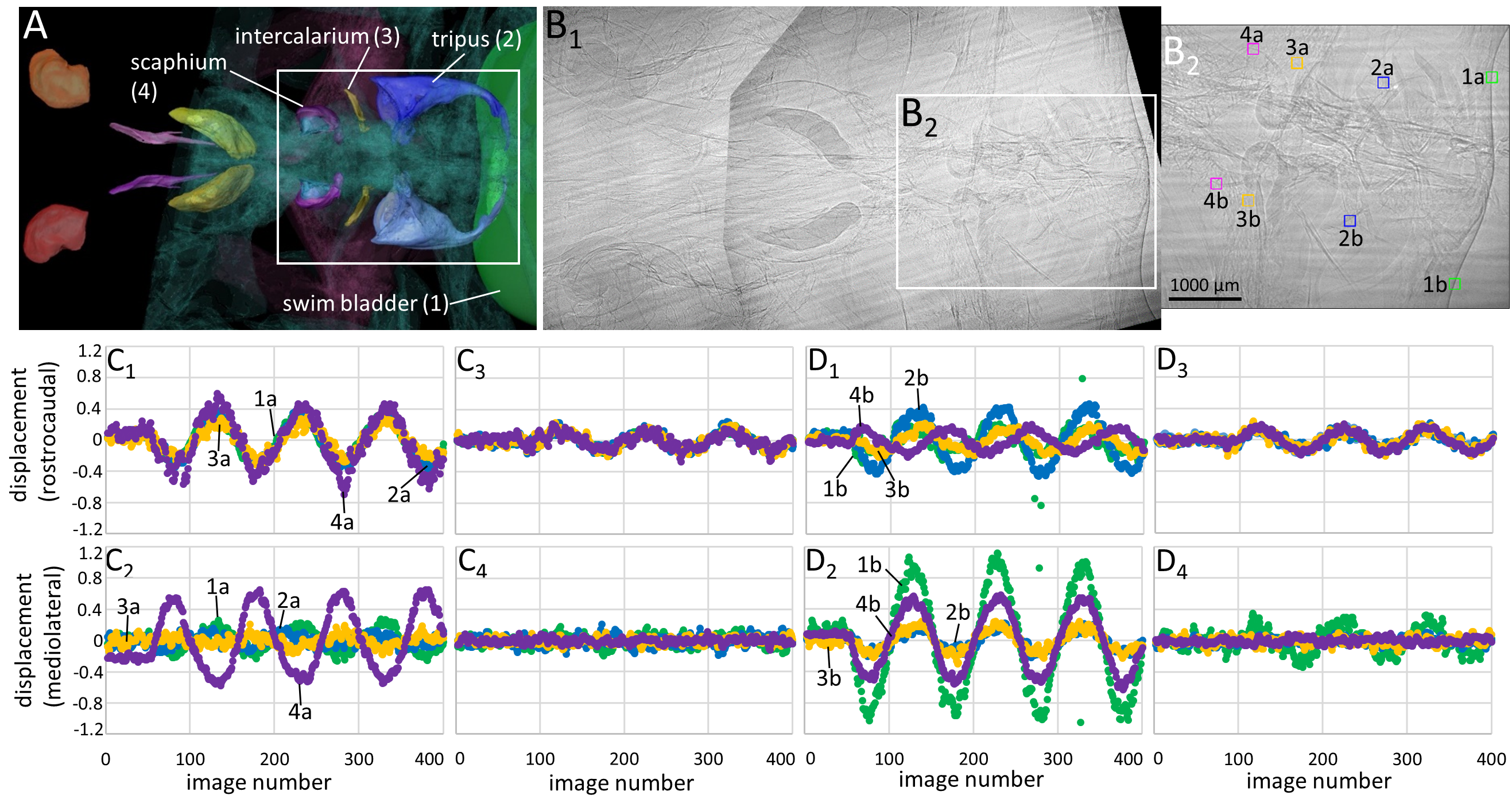

Supplement: S3 Fig — The fish (SL = 56 mm) was subjected to the in phase (0°, C1-C2, D1-D2) and the out of phase (180°, C3-C4, D3-D4) conditions (pixel size 3.67 µm, ID19). (A) 3D reconstruction of the structures shown in the 2D radiograph in (B1). (B1-2) “Landmarks” (squares of 40 × 40 pixels) depict the motion of the structures in x- (rostrocaudal) and y- (mediolateral) direction on the right (C) and left (D) body side during sound presentation. The Weberian ossicles and the anterior swim bladder wall show distinctly less displacement when the inertial shakers are driven out of phase (C3-C4, D3-D4) versus in phase (C1-C2, D1-D2). (TIF) [file pone.0230578.s010.tif]

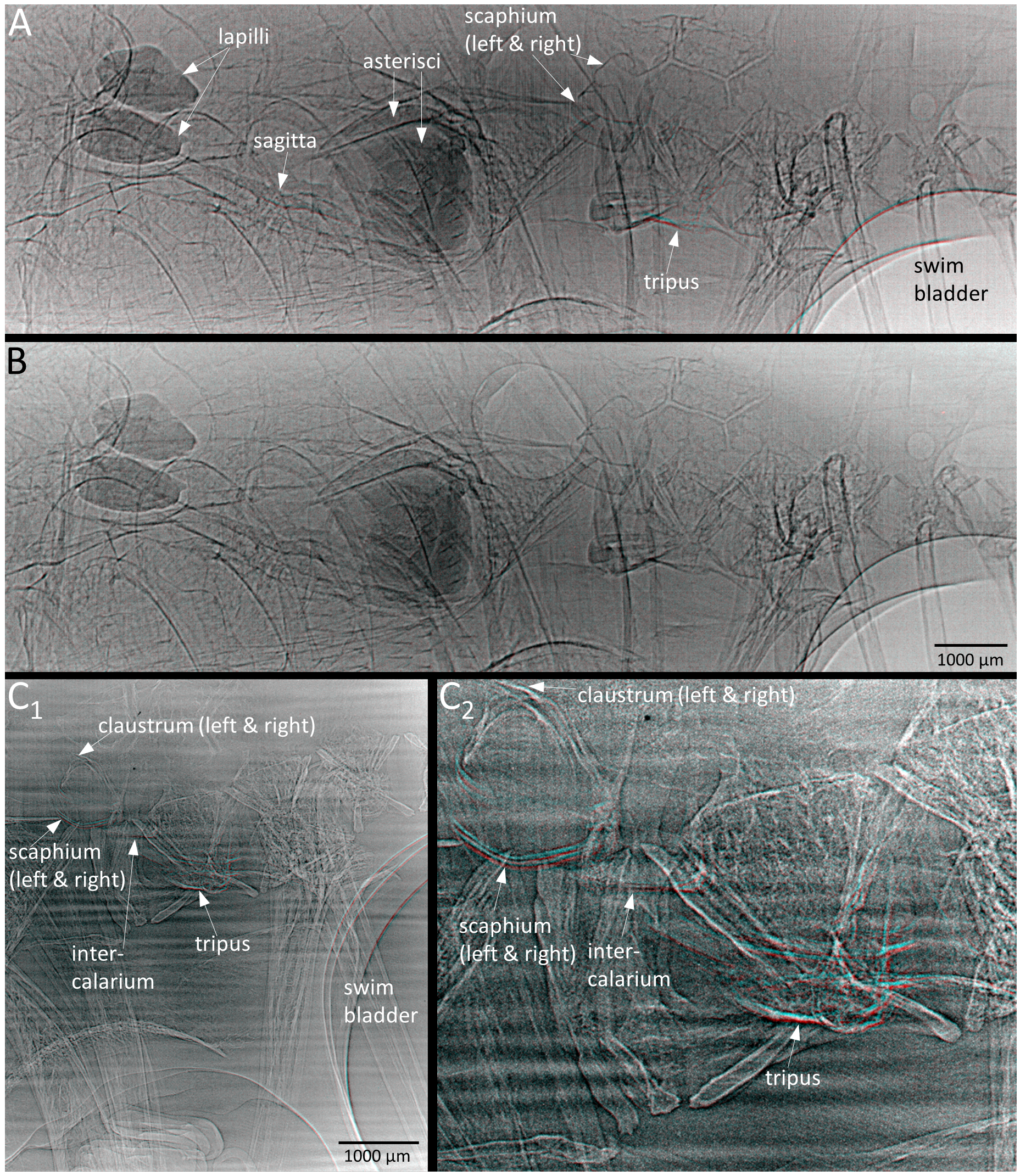

Supplement: S4 Fig — Overlays of averaged maximum (red) and minimum (green) positions indicate the motion of otoliths (sagittae), Weberian ossicles, and the walls of the anterior swim bladder portion due to stimulus presentation. (A) and (C) depict the structures during the in phase (0°) condition of the sound stimulus, (B) during the out of phase (180°) condition. (C2) illustrates that tripus, intercalarium, and scaphium move distinctly while the motion of the claustrum is weak. Structures outlined in red and green indicate motion, whereas uniformly grey, white, or black structures point to no or weak movement during sound presentation. (A-B) represent an individual (SL = 59 mm) studied at ID17 (pixel size 6.1 µm, frame rate 98.9954 fps) and C illustrates a specimen (SL = 50 mm) imaged at ID19 (pixel size 3.67 µm, frame rate 198.02 fps). (TIF) [file pone.0230578.s011.tif]

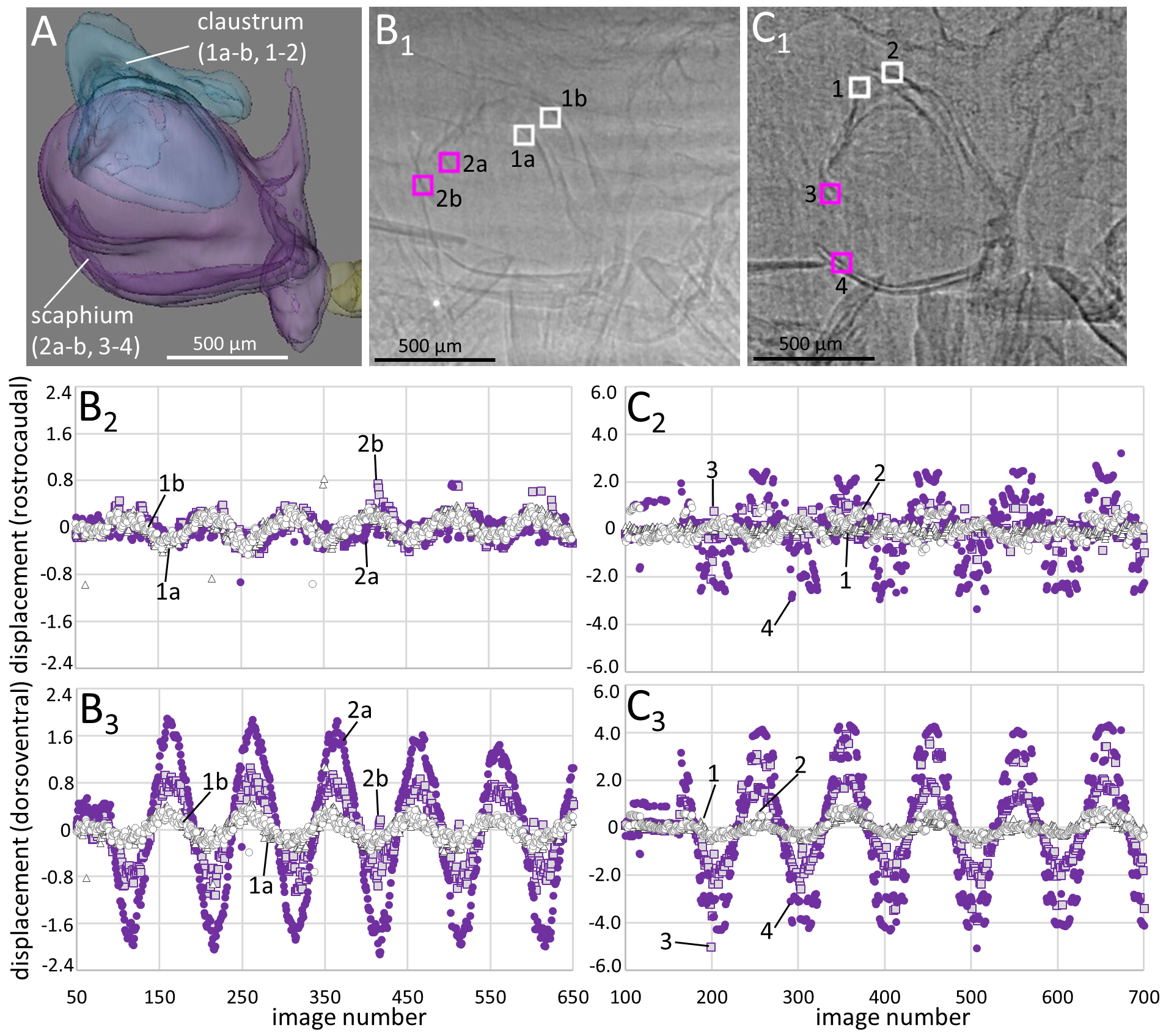

Supplement: S5 Fig — The fishes (B, SL = 50 mm; C, SL = 58 mm) were subjected to the in phase (0°) condition. (A) 3D reconstruction of the structures shown in the 2D radiograph in (B1) and (C1). In (B1, C1), “landmarks” (squares of 20 × 20 pixels) depict the motion of the structures in x- (rostrocaudal) and y- (dorsoventral) direction during sound presentation. Along the rostrocaudal axis (B2, C2), both ossicles show a similar amount of displacement (except landmark 4 in C2). Along the dorsoventral axis (B3, C3), the scaphium in both individuals is displaced more than the claustrum. (TIF) [file pone.0230578.s012.tif]

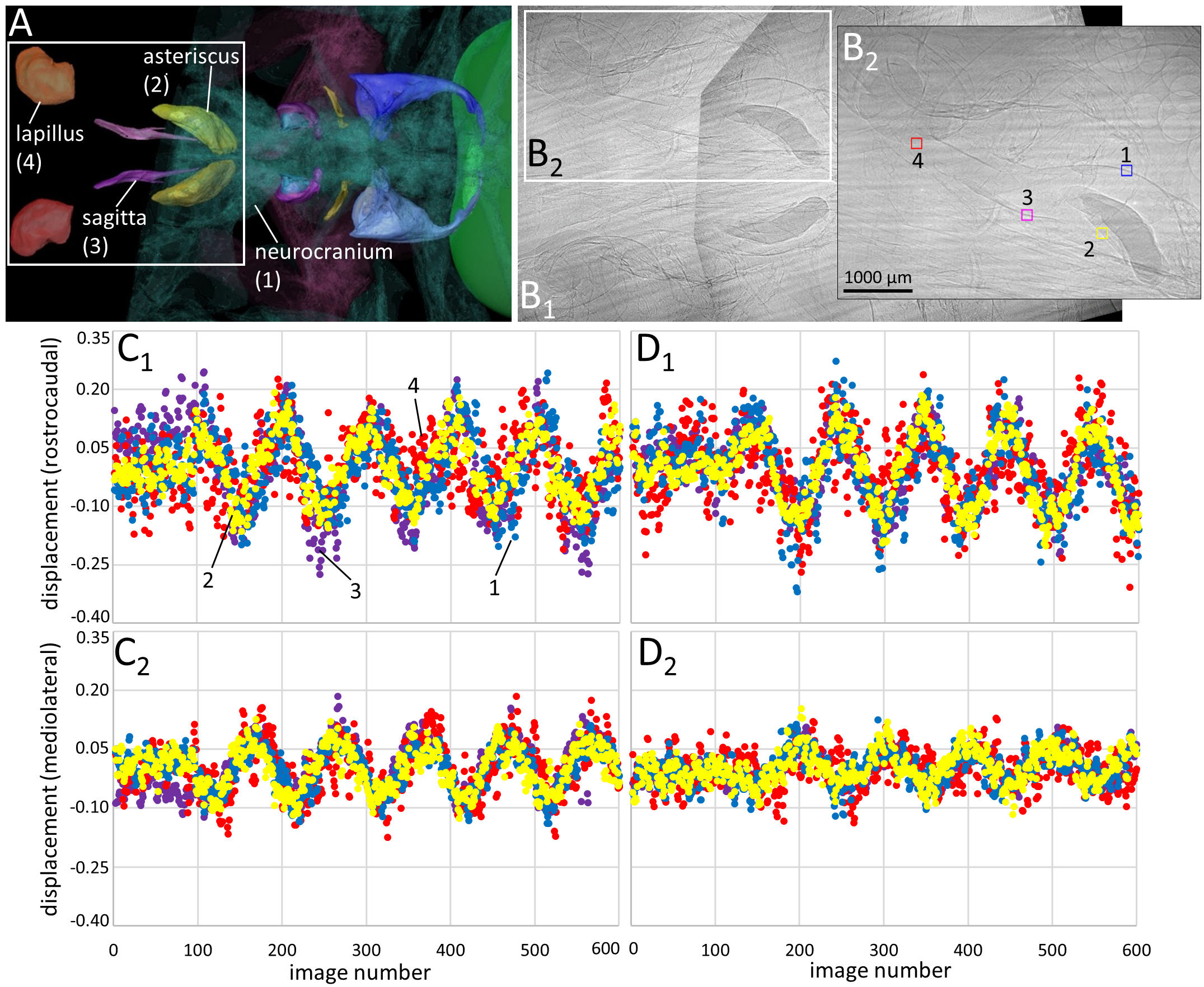

Supplement: S6 Fig — The fish (SL = 56 mm) was subjected to the in phase (0°, C1-C2) and the out of phase (180°, D1-D2) conditions (pixel size 3.67 µm, ID19). (A) 3D reconstruction of the structures shown in the 2D radiograph in (B1). (B1-2) “Landmarks” (squares of 40 × 40 pixels) depicted the motion of the structures in x- (rostrocaudal) and y- (mediolateral) direction during sound presentation. Otoliths show a greater maximum displacement along the rostrocaudal than along the mediolateral axis. The maximum displacement of lapilli and asterisci is similar regardless of whether the inertial shakers are driven in or out of phase. (TIF) [file pone.0230578.s013.tif]

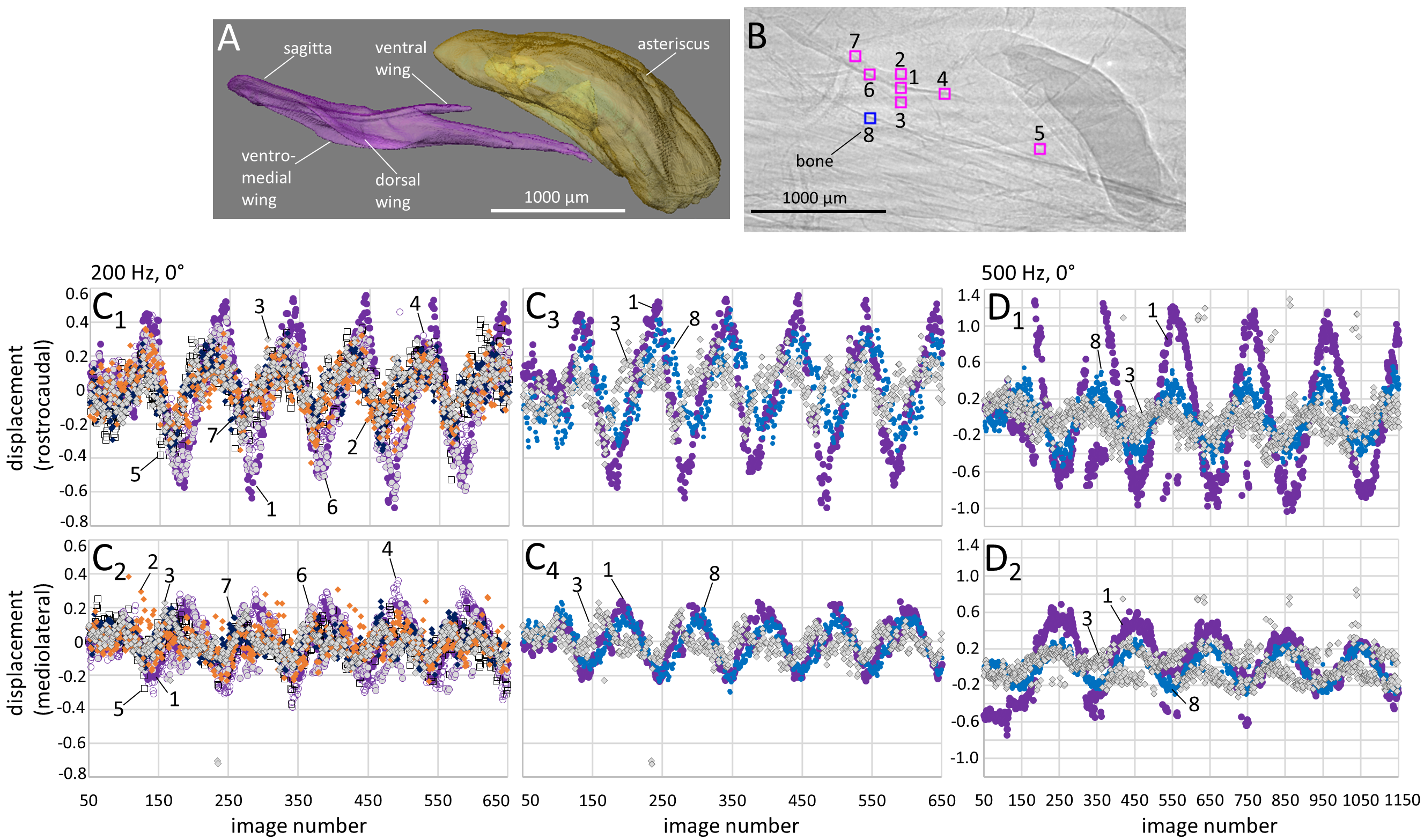

Supplement: S7 Fig — The fish (SL = 56 mm) was subjected to the in phase condition (0°) using a 200 Hz (SPL = 177.2 dB re1 µPa; frame rate = 198.02 fps; C) or a 500 Hz pure tone stimulus (SPL = 177.3 dB re 1 µPa; frame rate = 497.512 fps; D) (pixel size 3.67 µm, ID19). (A) 3D reconstruction of the structures shown in the 2D radiograph in (B). (B) “Landmarks” (squares of 20 × 20 pixels) depict the motion of the structures in x- (rostrocaudal) and y- (mediolateral) direction during sound presentation. In (C1-C2), landmarks 1, 4, and 6 show a phase shift with regard to landmarks 2–3 and 5–7, indicating a tilting motion of the sagitta. In (C3-C4) and (D1-D2) this phase shift is shown for the landmarks 1 and 3 also with respect to the motion of the adjacent bone (landmark 8). (TIF) [file pone.0230578.s014.tif]
