# Supplementary material for: Auditory chain reaction: Effects of sound pressure and particle motion on auditory structures in fishes
Source: PLoS One. 2020 Mar 27;15(3):e0230578. doi: 10.1371/journal.pone.0230578 (PMC7100961; doi:10.1371/journal.pone.0230578)
Supplement: Attachment — Submitted filename: Response to Reviewers 17022020.docx [file pone.0230578.s015.docx]

**Response letter – Ms-ID: PONE-D-19-30473**

**Reviewer #1:**

The manuscript is a very well written paper that investigates sound-induced motion of the otoliths within the fish inner ear end organs from two fish that have different otophysic connections of the swim bladder to the inner ear. This paper is a follow up study to their previous work but in the current paper the authors a standing wave tube-like tank to examine the motion of the inner ear otoliths under conditions where there is maximum particle motion vs maximum sound pressure. The authors provide the first experimental evidence of how the two different types of otophysic connections affect otolith motion in the fish inner ear. The subject of the paper should be of general interest to the readers of Plos One. I have only a few comments below.

**Thank you very much for the positive feedback on our ms.**

1) The stimulus used to induce motion of the otoliths within the end organs was quite high ranging from 141 to >177 dB re 1 microPa. The authors reported (in the methods lines 206-207) that “step (at ID19) equals a SPL of 177.2 dB re 1 microPa when shakers were in phase and a SPL of 155.4 re 1 microPa when shakers were driven out of phase”. These sound levels seem to be out of the biologically relevant ranges. I realize that these levels were likely required in order to visualize the induced motion of the otoliths. My concern is that the induce motion and how it may activate motion of the inner ear may not be biologically relevant to natural auditory stimuli experienced by these fish in nature. To alleviate such concerns, the authors should at least address this issue and explain the limitations (I know they have to some degree in the discussion) of how more natural sound levels might induce similar motions at lower levels.

**We address this point in the Discussion to a larger extent than previously. We completely agree that the high SPLs used in our study are of no biological significance but they are necessary to detect otolith oscillations in situ using hard X-ray imaging. Spatial and temporal resolutions at tomography beamlines are constantly improved. Hence, it may be possible to characterize otolith motion at much lower SPLs and higher frequencies in future studies.**

2) An interesting note on line 399-401 that states that the left and right Weberian ossicles revealed differences in the amount of displacement when subjected to sound induced motion. The authors also describe the differential motion of the otoliths based on experimental condition. Are the authors suggesting that the Weberian apparatus might afford directional sensitivity to the sound source? This would be extremely interesting if true. Also, their description of the sound induced movement of the otoliths in the Canara pearlspot cichlid may have implications for how the inner ear uses the particle motion and indirect sound pressure cues to determine sound source direction via the phase model hypothesis.

**The different motion patterns of otoliths depending on experimental conditions might have implications for sound source localization. However, we refrained from discussing such a possibility because the topic of explaining the mechanisms of how fish may be able to unambiguously localize a sound source is still vividly debated. As we used high SPLs and as our setup cannot provide a 100% separation of sound-induced particle motion and sound pressure, we felt it being too speculative to interpret our data with regard to sound source localization.**

3) The authors also describe other interesting results of the “tilting motion” of the sagitta in E. canarensis. Was there any evidence for the so-called “rocking motion” of otoliths under their experimental conditions as described by Krysl et al (2012) in PloSOne.

**Based on our 2D radiographic imaging, it is difficult to decide whether or not otoliths such as the sagitta in *E. canarensis* may show a “rocking motion”. Under these imaging conditions, we observe a moving auditory structure only from a certain view often overlain by other skeletal elements like cranial bones. Capturing the motion of the otoliths in their full 3D aspect in future experiments using tomography is likely to allow for a more precise characterization of the motion pattern and may clarify whether the movement is a rocking motion *sensu* Krysl and colleagues.**

4) Surprisingly the authors do not discuss the induced movement of the asteriscus in E. canarenis given the close proximity of the lagena to the swim bladder in the cichlid. Although sound induce movement was minimal at the experimental sound levels used, movement of this otolith could be very important in terms of the indirect detection of particle motion from the swim bladder. Fay reported that fish can detect particle motion displacement as low as 1 nm. Thus, the authors should not discount the importance the lagena and saccule given their close proximity to the swim bladder.

**We agree that the close proximity of the lagena to the swimbladder extensions in *Etroplus* may indicate a more prominent role in audition of this otolith end organ. However, due to a rather weak contrast of asterisci compared to sagittae and lapilli (see please Fig. 6B_1_ vs. A) – we could hardly identify the asterisci in the radiographs when the fish was seen in lateral and frontal views – in combination with a weak overall motion of asterisci, it was very difficult to characterize the motion pattern of the lagenar otoliths. Hence, we think that our data do not allow for discussing or interpreting the motion of the lagenar otoliths.**

**Reviewer #2:**

The study using a new apparatus to image the auditory portions of the inner ear during both sound pressure and particle motion. The design is clever and is novel in that it could image this structures during stimulation. The authors do a good job of indicating potential limitations with their procedures. The study makes a very nice overall contribution to the field.

**Thank you very much for the positive feedback on our ms.**

A couple of items that need to be clarified or considered.
The fish are dead and tissue structure can break down very quickly, especially if high intensity lighting was needed and it generated heat. It would have been useful if there was postmortem examination to determine if there were any structure changes that could not be detected by imaging. For example, what was the effect of blood clotting?

**We would like to refer to our statement made in the Material and methods section:**

**“To minimize detrimental effects of X-ray-induced tissue damage, experiments performed in the same orientation (e.g. fish seen in lateral view) and using the same species, but different individuals, alternately started with the in phase (0°) and the out of phase condition (180°; for an explanation of the two conditions see “setup design”). Hence, we started using the in phase condition and the highest studied sound pressure level (SPL; step 1, Table 1) in one specimen and using the out of phase condition at a lower SPL (e.g. step 4, Table 1) in another individual of the same species.”**

**In the discussion of the revised version of the ms, we included a short statement that future studies should compare tissue damage on the histological level by comparing control fish with specimens subjected to hard X-ray imaging.**

The SPL were very high and not normally used in fish auditory studies. Was this due to the limitations in the imaging? I would expect to see large scale movements at these SPL that fish may never experience. How did this impact the studies and models?

**Yes, this is exactly the point that we had to find a compromise between the acoustics and the constraints implied by the imaging procedure to obtain a meaningful signal-to-noise ratio. We had to go for rather high SPLs because the currently achievable spatial and temporal resolution is not high enough to visualize otoliths moving only a few nanometers as would be expected for lower SPLs (e.g. at the auditory threshold). We agree that this is an important aspect and we thus briefly address this issue in the discussion in the revised version of the ms.**

I presume the test chamber is designed to get around the problems associated with auditory studies in small tanks. Can physiological studies eventually be done in these types of tanks?

**We think that this is a very interesting aspect. It may be feasible to measure e.g. AEPs during sound stimulation while imaging the moving auditory structures, if it was possible to subject live animals to an acceptable X-ray dosage while still yielding a good signal-to-noise ratio.**

I felt the discussion is far too speculative. The authors have a very nice data set and do not need to explain every variance that may be impacting the structure. I would suggest that the discussion focuses on what can definitely be determined and limit the number of speculative discussion.

**As recommended, we shortened the discussion, especially with regard to potential applications of the setup.**

I realize this is an on line journal and page limits are not a concern, however 18 figures seem a little excessive and it is hard for the reader to wade through and determine which ones are really need. I would suggest that several figures could be moved to supplemental files.

**As suggested, we reduced the number of figures in the main text to 11 instead of 18 and moved 7 figures to the Supporting information.**

Minor
Abstract The use of the term maximum even in quotations is confusing.

**Corrected as suggested. We rephrased these sentences using “maximised” instead of “maximum”.**

29 “inner” ears. Many times the authors use the term ear, when inner ear would be more accurate

**Corrected as suggested. We now use “inner ears” instead of just “ears” throughout the manuscript.**

40 I think it is safe to say they are no longer “assumed”

**Corrected as suggested. Now it reads “…and play an important role…”**

45-46. Sentences end and start with same words

**We rephrased this sentence to avoid at least part of the repetition. It reads now as follows: “These structures….”**

What is an improved hearing ability. This is usually termed increased sensitivity or greater frequency range

**We rephrased the half-sentence to be more precise/specific: “….which are often correlated with a widened range of detectable frequencies (especially at frequencies > 700 Hz) and increased auditory sensitivity.”**

51 … whole body in motion: It is unclear the effect of hard skeletal elements. Also, the motion is oscillolatory with no net movement gain and therefore motion is not best word? Vibration?

**We rephrased this accordingly: “….results in a to and fro motion of the fish..” to make clear that it is not a net movement into a certain direction.**

78 The pearlspot is not considered as well investigated as the goldfish

**We agree that goldfish and *E. canarensis* are not equally well investigated with regard to the auditory structures and auditory abilities. We therefore rephrased this part as follows:**

**“Goldfish is well investigated with regard to inner ear and swim bladder morphology. For *Etroplus*, data on the gross morphology of the auditory structures are available.”**

95 The saccule has plays a very important vestibular function and therefore does not “mainly” function in audition

**We corrected the sentence accordingly:**

**“1) In otophysans such as goldfish, the saccule is assumed to play an important role in perceiving sound pressure.”**

113 The cessation of opercular movement is not considered an endpoint of euthanasia. Many fish (including goldfish) can be easily revived after the cessation of opercular movement. There should have been an additional period following the cessation of movement to insure complete euthanasia

**As we used a strong solution of the anesthetic (0.4% buffered MS-222), opercular movements ceased rather quickly, i.e. within 5 minutes. After that we waited further 10-15 minutes before further handling of the fish. In the revised version of the ms, we clarified this accordingly.**

Methods
The image rate of 200 Hz for the 200 Hz seems relatively slow. Most video is run higher than stimulus. Please explain if this had an effect on resolving the movements

**We would like to refer to a section in the original version of the ms addressing this issue: “From an imaging point of view, the “large” water body in the tank impairs a real-time observation of the moving auditory structures in the fish. At ID19, maximum image acquisition rates of up to 1 kHz (1,000 fps) are possible, which would be sufficient to record these motions in real time. However, such high frame rates are only possible for samples investigated in air [60,61]. It is thus important to note that our observations of moving otoliths and ancillary auditory structures are a proxy of the true motion.”**

633 add reference

**Corrected as suggested. We included the respective reference, i.e. Vetter et al. (2019) J Comp Physiol A.**

Table 1: delete, add info to text

**As recommended, we deleted Table 1 and included the information in the text.**
